# HOX and PBX gene dysregulation as a therapeutic target in glioblastoma multiforme

**DOI:** 10.1186/s12885-022-09466-8

**Published:** 2022-04-13

**Authors:** Einthavy Arunachalam, William Rogers, Guy R. Simpson, Carla Möller-Levet, Gemma Bolton, Mohammed Ismael, Christopher Smith, Karl Keegen, Izhar Bagwan, Tim Brend, Susan C. Short, Bangxing Hong, Yoshihiro Otani, Balveen Kaur, Nicola Annels, Richard Morgan, Hardev Pandha

**Affiliations:** 1 Targeted Cancer Therapy, Department of Clinical and Experimental Medicine, Faculty of Health and Medical Sciences, University of Surrey, Guildford, GU2 7WG UK; 2 Surrey Technology Centre, HOX Therapeutics Ltd, Unit 2440 Occam Rd, Guildford, GU2 7YG UK; 3 Department of Pathology, Royal Surrey County Hospital, Egerton Road, Guildford, GU2 7XX Surrey UK; 4 Faculty of Medicine and Health, Leeds Institute of Medical Research at St James’s, University of Leeds, Leeds, LS9 7TF UK; 5 Department of Neurosurgery, McGovern Medical School, University of Texas Health Science Centre at Houston, 7000 Fannin Street, Houston, TX 77030 USA; 6 School of Biomedical Sciences, University of West London, St Mary’s Road, Ealing, London, W5 5RF UK

**Keywords:** Glioblastoma multiforme, HOX, PBX, Dysregulation

## Abstract

**Background:**

Glioblastoma multiforme (GBM) is the most common high-grade malignant brain tumour in adults and arises from the glial cells in the brain. The prognosis of treated GBM remains very poor with 5-year survival rates of 5%, a figure which has not improved over the last few decades. Currently, there is a modest 14-month overall median survival in patients undergoing maximum safe resection plus adjuvant chemoradiotherapy. *HOX* gene dysregulation is now a widely recognised feature of many malignancies.

**Methods:**

In this study we have focused on *HOX* gene dysregulation in GBM as a potential therapeutic target in a disease with high unmet need.

**Results:**

We show significant dysregulation of these developmentally crucial genes and specifically that *HOX* genes A9, A10, C4 and D9 are strong candidates for biomarkers and treatment targets for GBM and GBM cancer stem cells. We evaluated a next generation therapeutic peptide, HTL-001, capable of targeting *HOX* gene over-expression in GBM by disrupting the interaction between HOX proteins and their co-factor, PBX. HTL-001 induced both caspase-dependent and –independent apoptosis in GBM cell lines.

**Conclusion:**

In vivo biodistribution studies confirmed that the peptide was able to cross the blood brain barrier. Systemic delivery of HTL-001 resulted in improved control of subcutaneous murine and human xenograft tumours and improved survival in a murine orthotopic model.

**Supplementary Information:**

The online version contains supplementary material available at 10.1186/s12885-022-09466-8.

## Background

Glioblastoma (GBM) is the most common and most aggressive of all malignant brain and central nervous system tumours. It is characterized by uncontrolled cellular proliferation, high vascularity, increased necrosis and diffuse brain infiltration [1, 2]. The prognosis is poor, only 2% of GBM patients aged over 65 years, and 30% of patients aged under 45 years at diagnosis survive ≥ 2 years [3]. The five-year survival rate of GBM is a dismal 5%, with a median survival of 15–17 months [4]. Little improvement has been made in treatments for GBM over the past 4 decades. The standard of care is surgical resection followed by radiotherapy and adjuvant oral temozolomide (TMZ) [5, 6]. The median survival time for patients receiving adjuvant temozolomide and radiotherapy is 15 months (5). These statistics highlight the urgent need for new and effective therapies.

The many challenges of treating GBM include drug delivery to the tumour site, hampered by the presence of the blood–brain barrier (BBB) [4, 7]. GBM effectively also has its own BBB due to abnormal neovasculature with irregular blood flow further preventing drugs from exiting the circulation, which, in turn, influences the treatment of the tumour when drugs are delivered systemically [8]. Other factors that contribute to a poor prognosis are tumour cell migration into the surrounding tissue, immune evasion [8], and evasion of cell death induced by radiation and chemotherapy through the activation of anti-apoptotic resistance pathways and upregulation of DNA repair systems. In addition, GBMs are often cancer stem cell (CSC) enriched, possibly due to their close proximity to the ventricles of the brain which are stem cell production and maturation sites [9, 10]. CSCs are vital to the continued growth of the tumour as they have an ability to self-renew, proliferate and form differentiated cancer cells. Targeting of CSCs may be essential for successful GBM treatment as they are associated with resistance to conventional treatment, including radiation and chemotherapy [11].

The *HOX* genes encode a family of homeodomain-containing transcription factors that play important roles in the early embryo, including the establishment of cell and tissue identity, and the regulation of cell proliferation, differentiation, and survival [12]. They are organised into four clusters, A, B, C and D, each of which is located on a different chromosome [13]. The highly conserved homeodomain of HOX proteins mediates their binding to DNA, although the strength and specificity of this interaction is greatly increased by the binding of co-factors such as Pre-B-cell Leukaemia Homeobox (PBX), which forms heterodimers with HOX proteins in groups 1–9, and Myeloid Ecotropic Viral Integration Site 1 Homolog (MEIS) proteins that dimerize with HOX proteins 9–13 [14]. These cofactors have a role in the recruitment of RNA polymerase II or III, as well as transcriptional inhibitors such as histone deacetylase (HDAC), resulting in differential gene regulation depending on the sequence and context of the target site in the enhancer or promoter region [15, 16]. Many of the *HOX* genes are over-expressed in a range of cancers including GBM [17], melanoma [18], and head and neck [19], prostate [20], breast [21], ovarian [22], and pancreatic cancer [23]. In GBM, studies of specific HOX gene dysregulation suggests overexpression of the HOXA cluster to be most prevalent, possibly due to gain of additional copies of chromosome 7 that harbours this cluster, and activation of the PI3K/AKT pathway [24]. *HOXA9* expression was shown to be predictive of poor GBM patient outcome and associated with pro-proliferative and anti-apoptotic functions [25]. However, comprehensive studies of all 39 HOX genes in normal brain tissue, GBM and GBM CSCs are lacking.

The key roles that HOX and PBX proteins play in cancer indicate that they are potential therapeutic targets. However, the high level of functional redundancy amongst HOX proteins and the general difficulty in producing effective small molecule inhibitors against transcription factors have proved significant barriers to this approach. As an alternative, it was proposed that the interaction between HOX and PBX proteins could be targeted, as this is mediated by a highly conserved hexapeptide sequence in HOX proteins and a hydrophobic binding pocket within PBX [26]. To date, a more useful set of inhibitors have proved to be peptides that employ the hexapeptide sequence to act as a competitive antagonist of HOX/PBX binding. Several peptides have been described, but the one most frequently used is HXR9, an 18 amino acid peptide containing the hexapeptide sequence together with 9 arginine residues that promote cellular uptake by endocytosis. HXR9 was originally shown to be cytotoxic to melanoma cell lines and subsequently to a broad range of solid and liquid cancers (reviewed in [27]).

In this study, we investigate the nature and extent of *HOX* gene dysregulation in GBM in human cell lines, cell line-derived CSC and patient tissue. We evaluated a next generation peptide therapeutic, HTL-001, employing the hexapeptide sequence to act as a competitive antagonist of HOX/PBX binding, capable of rapidly inhibiting HOX/PBX dimer formation, and triggering significant anti-tumour effects.

## Methods

### Cell lines and primary tissue

Cell lines GL261, U87-MG, A549 and HT29 were purchased from the American Type Culture Collection (ATCC) and U251-MG and LN18 from the European Collection of Cell Culture (ECACC). Cell lines KNS42, SF188 and RES186 were a kind gift from Professor Chris Jones (Institute of Cancer Research, UK) and GBM4 by Dr Heiko Wurdak (University of Leeds, UK) respectively. Commercially available cell lines were authenticated using short tandem repeat (STR) profiling (LGC, UK), and compared to authenticated STR profiles, with a threshold of ≥ 80% confirmed as a match.

All cell lines were adherent lines, cultured in a Nuaire In-VitroCell incubator (Nuaire, USA) at 37 °C with 5% CO_2_, and 25% O_2_. Mycoplasma testing was carried out prior to and after cryopreservation, and regularly thereafter on all cell lines using MycoAlert**™** Mycoplasma Detection Kit (Lonza, UK). Brain tissue from healthy children was obtained from an existing program at the National Institute of Health (https://neurobiobank.nih.gov/).

### Bioinformatic analysis

RNA-seq gene expression quantification for TCGA-GBM was downloaded from the TCGA repository (https://portal.gdc.cancer.gov/repository) on 9^th^ October 2018. Gene expression comparisons between 154 primary solid tumours and 5 independent solid normal samples were based on the R package EdgeR (v3.24.3). Lowly expressed genes were filtered out by keeping genes with Counts Per Million (CPM) > 0.165 (median CPM of 8 counts) in at least 5 samples. *HOXB1* did not pass the expression filtering threshold. Data was normalised using the trimmed mean of M-values normalisation method (TMM) [28]. A negative binomial generalized log-linear model was fitted to the read counts for each gene and likelihood ratio tests for tumour vs normal tissue differences were conducted. GBM microarray expression data were obtained from Lee et al. 2006 [29] (22 GBMs and 3 normal neural stem cell samples) GEO accession number GSE4536; and Sun et al. 2006 [30] (81 GBMs and 23 normal brain samples) GEO accession number GSE4290. The summarised expression data obtained from GEO was log2 transformed and median-centred normalised. The t-test statistic was used to evaluate differential expression between tumour and normal samples. In all analyses *p* values were adjusted for multiple comparison using the Benjamini and Hochbrg (BH) approach [31]. The name of the clinical variables used for the survival analyses are: days_to_death, vital_status and days_to_last_follow_up. Out of the 154 primary solid tumour samples, two samples had no time of dead or time to last follow up, leaving 152 samples. All survival analyses were performed using the R packages survival (3.2–7) and survminer (v 0.4.9) on TMM normalised log2(CPM). Each gene was assessed through a univariate Cox regression model and overall survival Hazard ratios (with 95% confidence interval) were calculated using stratified gene expression (high = above 75% and low = below 25%). Kaplan–Meier overall survival analyses of patients stratified according to gene expression (high = above 75% and low = below 25%) were performed and log rank p-values calculated.

### MTS cell survival, Annexin V / 7-AAD, caspase 3/7, western blotting and RT-qPCR assays

In vitro assays for cell survival, apoptosis, and gene expression were performed using standard methodologies that have been previously described. Full details are given in Appendix A.

### Animal studies

All murine experiments were performed in accordance with and approval of UK Home Office and University of Surrey, and Animal Welfare Committee (AWC) at University of Texas Health Science Centre.

### Subcutaneous models

100ul of live U87-MG cells (1 × 10^6^ cells) in 50% matrigel/50% Hanks was injected SC in the right flank of the animals (n = 8 per group). When tumours were between 80-90mm^3^ in volume, the animals received an injection at a single site (IP), a maximum of three times in a week (1–2 day apart), for three consecutive weeks, of HLT001 or PBS. GL261 subcutaneous tumors were established as previously described [32]. Mice were treated when the tumor reached 100 mm^3^ and randomized into groups receiving an inactive control peptide (CXR9) or HTL-001(30 mg/kg) i.p. three times a week until experiment end. CXR9 is an identical structure to the first generation HXR9 agent, except has a substitution of an alanine for tryptophan which abrogates the PBX binding completely. HTL-001 or CXR9 was dissolved in PBS at 5 mg/ml. Statistical significance was evaluated by ANOVA with Satterthwaite’s approximation used to calculate the degrees of freedom. *p* value less than 0.05 was considered as significant. At the end of treatment, tumors were removed and placed in 4% paraformaldehyde and embedded in paraffin for IHC staining.

### Syngeneic intracranial glioma model

To induce intracranial tumors in C57BL/6 J mice, GL261 cells (1 × 10^5^) in a total volume of 2 ul were injected into the striatum of mice by sterotactic injection as previously described [33]. Mice were then randomly assigned to control and treatment groups. 7 days after tumor inoculation, the tumor-bearing mice were i.p. treated with CXR9 or HTL001 (30 mg/kg) three times a week until experiment end. For intra-tumor treatment, 600 µg CXR9 or HTL001 in 5 µl PBS were injected into tumor. For HTL-001 Alexa 660 studies, 10 mg/kg HTL001-Alexa 660 were i.p. injected into tumor-bearing mice. 30 min later, mice were sacrificed, and organs were harvested for imaging using a Cy5.5 filter in the IVIS imaging system (Perkin Elmer) with exposure time of 10 s. Tumor growth was monitored using MRI with a 7 Tesla MRI scanner (Bruker Biospin, Billerica, MA). Kaplan–Meier analysis was used to estimate the survival over time and log-rank test was performed to test the statistical significance. After treatment, the brains were removed and processed for IHC staining.

### Intravital imaging

Six- to eight-week-old male or female NOD scid gamma **(**NSG) mice were used. GBM12-RFP cells (2 × 10^5^ cells) were implanted stereotaxically into the right hemisphere and craniectomy was performed 2 weeks later in which a cover glass a fixed to the skull. For intravital imaging, mice were anesthetized and positioned on the stage of a confocal microscope (NIKON), and pre-treatment images were acquired. Then, 100 µl of HTL-001-Alex 660 peptide (5 mg/ml in PBS) was administrated through intraperitoneal or tail vein injection. One hour later, 100 µl of 10% fluorescin isothiocyanate (FITC)-conjugated dextran (500 kDa, Sigma-Aldrich) was injected into the tail vein, and post-treatment images were acquired. To evaluate the accumulation of peptide in time-course, 100 µl of HTL-001-Alex 660 peptide (5 mg/ml in PBS) was administrated intraperitoneally on day 2, and the confocal images of same area were acquired on day 3.

### Co-localisation immunofluorescence

Cells were plated with 1 × 10^4^ cells per well in 8-well chamber slides with coverslips for 24 h at 37 °C. Cells were then treated with media or drug for 2 h. All wells were fixed in 3.7% paraformaldehyde for 15 min and permeabilized with 0.5% Triton X‐100 for 5 min. Wells were then blocked in 5% BSA and then incubated in primary antibody dilutions overnight at 4 °C, and then with the appropriate fluorophore‐conjugated secondary antibody dilutions (more details in Supplementary Methods section). Chambers were removed and slides were mounted and counterstained with SlowFade Gold AntiFade Mountant with DAPI (Thermo Fisher). FITC (green) and TRITC (red) emissions were confocally visualized by laser scanning microscopy (Nikon A1M Confocal Microscope and DS-Qi1 Widefield Camera).

## Results

### HOX gene expression

#### HOX genes are overexpressed in GBM tissues compared to normal tissues

**Fig. 1:**
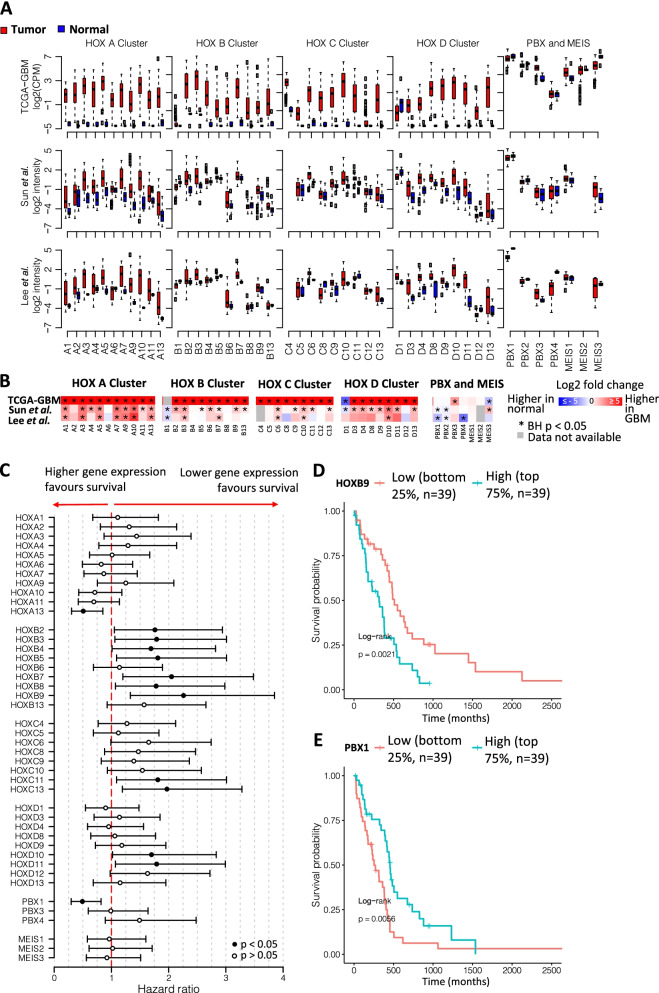
*HOX* genes expression in GBM tissues compared to normal tissues.** A** Expression levels of HOX genes and co-factors in the TCGA-GBM RNA-seq, Sun et al. 2006, and Lee et al. 2006 datasets. **B** Heatmaps of fold change values between GBM and normal. **C** Overall survival hazard ratios (with 95% confidence interval) of univariate Cox regression models using stratified gene expression. **D** Kaplan–Meier overall survival of patients stratified according to HOXB9 gene expression as high (above 75%) and low (below 25%). **E** Kaplan–Meier overall survival of patients stratified according to PBX1 gene expression as high (above 75%) and and low (below 25%)

While individual *HOX* gene dysregulation has been reported in GBM, [24, 25], few groups have assessed the expression of all 39 HOX genes and none have included assessment of their co-factors. The Cancer Genome Atlas (TCGA) provides unbiased real-world data from 156 primary GBM tumours and 5 normal brain samples. A total of 38 out of 39 HOX genes passed the expression filtering threshold and had a significantly different (BH *p* < 0.05) gene expression in GBM primary tumour compared to normal tissue. All dysregulated HOX genes had higher expression in GBM, except for *HOXD1*, which had higher expression in normal tissue (Fig. 1A and 1B). PBX and MEIS genes showed higher levels of expression and lower levels of sample variability within normal conditions compared to HOX genes (Fig. 1A). *PBX3* was significantly up-regulated (BH *p* < 0.05) in GBM, while *MEIS3* was significantly down-regulated. In addition to the TCGA GBM RNA-seq dataset, we examined the expression of HOX genes and co-factors in the Sun et al. 2006 [30] and Lee et al. 2006 [29] GBM microarray datasets (Fig. 1A and 1B). Although microarrays have a narrower dynamic range and can suffer from non-specific and cross-hybridization, overall, there was a good agreement with the TCGA GBM RNA-seq dataset. In Sun et al. 2006 [30] we found 30 HOX genes with significantly different (BH *p* value) gene expression in GBM compared to normal tissue, 28 genes up-regulated in tumour and, *HOXB1* and *HOXD1* down-regulated. Lee et al. 2006 [29] closely followed the expression profiles observed in Sun et al. 2006 [30], although having a smaller number of samples, a reduction in statistical power was expected. In this case, we found 12 HOX genes with significantly different (BH *p* < 0.05) expression in GBM compared to normal tissue, 11 genes up-regulated in tumour and *HOXD1* down-regulated. While relative expression levels of PBX and MEIS were conserved, fold changes varied both among the two microarray datasets and when compared to the RNA-seq data. The differences observed in fold changes were mostly due to deviations in expression patterns in the non-tumor samples (Fig. 1A), corresponding to brain tissue from epilepsy patients in Sun et al. 2006 and normal neural stem cells in Lee et al. 2006. Around 30% of the 43 genes analysed showed a significant association with overall survival (Wald and log-rank *p* < 0.05). Out of the 13 significant genes, 11 showed an adverse prognosis with increased gene expression, while 2 genes showed a favourable prognosis with increased gene expression (Fig. 1C-E).

#### HOX gene expression in human and a murine GBM cell lines

All 39 *HOX* genes were evaluated for RNA expression using RT-qPCR in normal human astrocytes (NHA), 3 human and 1 murine GBM cell lines. We found low or undetectable levels of *HOX* expression across all 39 genes in NHA. There was no discernible pattern of *HOX* expression common to all cell lines, nor was there a single predominant *HOX* gene or *HOX* cluster that was universally expressed by all cell lines. All tumour derived cell lines showed elevated levels of *HOX* gene expression across all 4 clusters (Additional Fig. 1A-C). In particular, high expression of the *HOXA* and *HOXD* clusters are the most pronounced in all cell lines. LN18 had a more dominant posterior *HOXA* expression pattern. U87-MG showed high central *HOXC* cluster and high posterior *HOXD* cluster expression. LN18 showed the highest expression of all 39 HOX genes compared to U87-MG and U251-MG. However, *HOXA3*, *HOXA5*, *HOXB9*, *HOXC4* and *HOXD9* showed the most consistent over expression across all parental and CSC cell lines. All these genes are expressed at higher level than that seen in NHA. HOX gene expression was compared between normal mouse brain and the murine cell line GL261 (Additional Fig. 1D). In normal brain, there was minimal expression apart from HOXA5. In GL261, the expression pattern was very different from those exhibited by human cell lines and showed upregulation of a number of HOXA genes (HOX A3,4,5,6,10,13), C10 and D8. These data highlight the high extent of *HOX* gene dysregulation in GBM and not in normal brain.

#### HTL-001 induces cell death in a panel of GBM cell lines

**Fig. 2:**
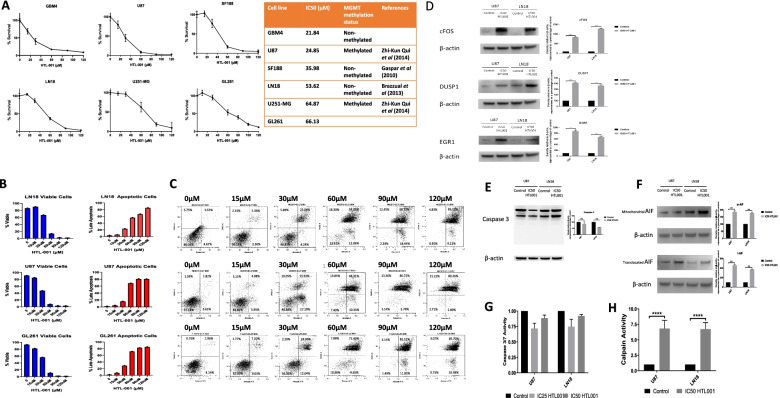
**A** *In vitro* assays demonstrate rapid and potent effect of HTL-001 in a panel of GBM cell lines. Cells were treated with HTL-001 for 2 hours at a range of doses (15, 30, 60, 90 and 120µM). The % cell survival was assessed relative to untreated cells via an MTS assay. HTL-001 IC50 values ranged from 21-84 to 66.13µM. Error bars represent standard deviation of 8 replicates within one biological repeat. **B** Annexin V and 7-AAD staining demonstrates that HTL-001 induces GBM cell death via apoptosis. The % viable cells, % apoptotic cells and corresponding FACS plots **C** (after Annexin V and 7-AAD staining) are shown in LN18, U87 and GL-261 GBM cell lines treated with HTL-001 for 2 hours at 15, 30, 60, 90 and 120µM. Error bars represent standard deviation within one biological repeat. **D** Total protein lysates were extracted from untreated and treated cells and probed with either an anti-cFOS antibody, an anti-DUSP1 antibody or an anti-EGR1 antibody, via western blot analysis. All three markers of interest showed significant upregulation in protein expression after 2-hour treatments with HTL-001. **E** Protein lysates were extracted from untreated and treated cells and probed with an anti-caspase 3 antibody via western blot analysis. Neither caspase 3 nor cleaved caspase 3 significantly increased after 2-hour treatments with HTL-001. **F** Subcellular fractionation was used to extract mitochondrial and nuclear protein lysates from cells treated with HTL-001. Western blot analyses with an anti-AIF antibody was used to show a significant increase in mitochrondrial and translocated AIF (mAIF and tAIF, respectively). **G** Caspase induction in cells treated with HTL-001 was measured by adding Caspase-Glo 3/7 assay reagent to respected wells and measuring the luminescence of triplicate wells. There was no significant increase in caspase 3/7 activity in cells treated with HTL-001. **H** Calpain induction in cells treated with HTL-001 was measured by adding Calpain-Glo Protease assay reagent to respective wells and measuring the luminescence of triplicate wells. There was a significant increase in active calpain present in cells treated with HTL-001, in comparison to the control level.

HTL-001 is a modified version of the original HOX/PBX peptide inhibitor, HXR9, previously shown to disrupt HOX/PBX dimer formation in a range of cancers (peptide sequences given in Appendix A). All the peptides used in this study contain a short polyarginine sequence that mediates rapid cell penetration by heparan sulphate proteoglycan-mediated endocytosis and nuclear localisation of the synthetic peptides [27]. In order to determine the in vitro potency of HTL-001, 6 adult GBM cell lines were treated with HTL-001 at a range of doses (15, 30, 60, 90 and 120 µM) for 2 h and then cell survival was assessed via an MTS assay, relative to untreated cells. We had previously determined there was no added effect of cell exposure longer than 2 h (data not shown). HTL-001 exposure led to a rapid anti-tumour effect in all 6 GBM cell lines tested, with IC_50_ values ranging from 21.84 µM to 66.13 µM (Fig. 2A). The most sensitive line was GBM4 (IC50 of 21.84 µM), a patient derived cell line, and the least sensitive the murine cell line GL261 (IC50 of 66.13 µM). There was no difference in sensitivity between MGMT methylated and unmethylated cell lines (Fig. 2A). HTL-001 was more effective in vitro than the prototype HXR9 peptide across GBM cell lines and cells lines from other malignancies (Additional Fig. 2).

#### HTL-001 induces GBM cell death via apoptosis

We investigated the mode of cell death for the GBM cell lines LN18, U87 and GL-261 induced by treatment with HTL-001 at 15, 30, 60, 90 and 120 µM for 2 h (Fig. 2B and C). Cells were analysed using flow cytometric analysis after labelling with the vital dye 7-Amino-actinomycin D (7-AAD) or Annexin-V-FITC. 7-Amino-actinomycin D can enter only dead cells, whereas Annexin-V-FITC recognises changes in the plasma membrane that are characteristic of apoptosis [34]. This revealed a dose-dependent increase in the percentage of cells undergoing late apoptosis when treated with HTL-001.

#### HOX gene dysregulation and targeting in GBM cancer stem cells

**Fig. 3:**
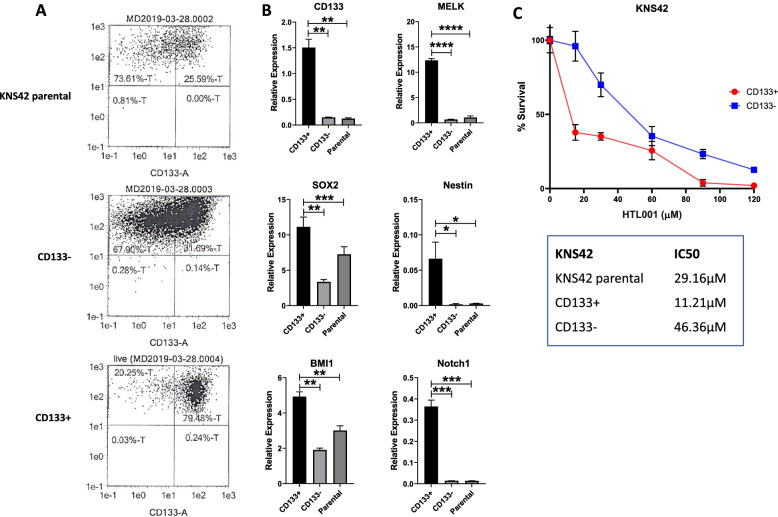
The KNS42 cancer stem cell population (CD133 positive cells) is more susceptible to HTL-001-induced cell death than KNS42 parental or CD133- cells. A. Staining/flow cytometric analysis showed that 79.48% CD133 + cells had been isolated. B. Cancer stem cell marker expression was determined in the three cell populations via Taqman qPCR. CD133, MELK, BMI1, SOX2 and Notch1 were all significantly upregulated in the CD133 + cell fraction compared to CD133- and parental populations. C. CD133 + cells (IC50 11.21 µM) were more sensitive to HTL-001 treatment than CD133- cells (IC50 46.36 µM) and KNS42 parental cells (IC50 29.16 µM). Error bars represent standard deviation within one biological repeat. Cell survival was analysed by non-linear regression: [inhibitor] vs response. – variable slope (variable parameters) and significance value was calculated using Two Way Anova Analysis with significance between the IC50 values being **. *P < 0.05, **P < 0.005 and ***P < 0.0005

The failure of current therapies to eliminate specific GBM stem cell subpopulations has been considered a major factor contributing to the inevitable recurrence in GBM patients following treatment. To investigate if a stem-like phenotype influenced response to HOX/PBX inhibition, we investigated the sensitivity of GBM CSCs to HTL-001 in the cell line KNS42, which has a stable sub-population of CD133 + stem cells, and neurosphere derived CSCs. KNS42 has significant HOX gene dysregulation but low expression of the A cluster compared to other GBM cell lines (Additional Fig. 2). CD133 + and CD133- cells were separated from the KNS42 parental cell line using microbeads, achieving an 80% CD133 + isolation (Fig. 3A). These cells were positive for stem cell markers (MELK, BMI1, SOX2 and Notch1), Fig. 3B, although, unlike the neurosphere derived CSCs, nestin expression was negative. Following 2 h exposure to HTL-001, CD133 + cells (IC_50_ 11.21 µM) were more sensitive to HTL-001 than CD133- cells (IC_50_ 46.36 µM) and KNS42 parental cells (IC_50_ 29.16 µM), Fig. 3C. We compared the sensitivity of HTL-001 in neurosphere-derived GBM CSCs grown in stem cell permissive conditions to that of their parental cell lines. Morphological and phenotypic parameters with respect to the stemness of the CSCs generated and shown in Additional Figure 4. We measured expression of all 39 HOX genes by RT-qPCR in parental GBM cells and their respective GBM CSCs. HOX A3, A5, B9, C4 and D9 showed the most consistent over expression across all parental and CSC cell lines (Additional Fig. 4A and B). CSC cell lines showed a further elevation of gene expression compared to their parental cell lines. CSC were also more sensitive to HTL-001 than parental cells (Additional Fig. 4C).

#### Specificity of HTL-001 targeting

**Fig. 4:**
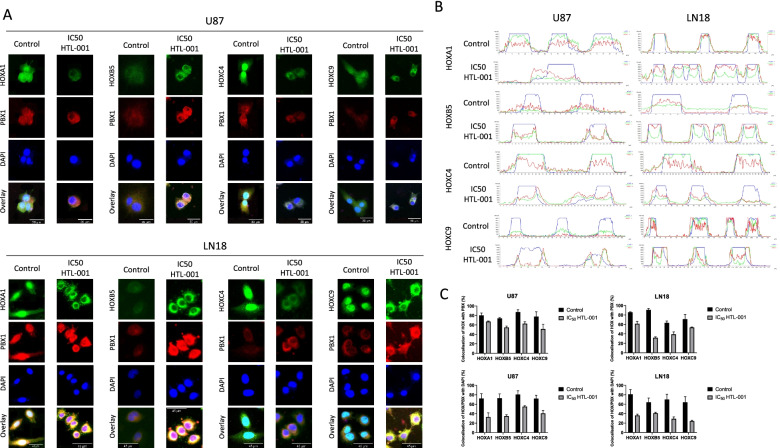
HTL-001 disrupts HOX-PBX interactions in glioblastoma cells. U87 and LN18 cells were treated with 25 µM and 54 µM HTL-001, respectively for 2 h. Cells were washed, fixed and permeabilised, after which they were stained with an anti-PBX1 antibody and an anti-HOX antibody (either against HOXA1, HOXB5, HOXC4 or HOXC9). Co-localisation of HOX and PBX were visualised for immunofluorescence by staining cells with fluorescence-labelled secondary antibodies against the primary antibodies, using confocal microscopy. **A** In HTL-001 treated cells, HOX-PBX dimer formation decreased significantly with the most significant decrease seen in the nucleus. **B** Intensity graphs were generated using NIS-Elements Confocal Software to visualise expression intensity of HOX (indicated by green lines), PBX (indicated by red lines) and DAPI for the nuclear region of the cell (indicated by blue lines). HTL-001 induced cytoplasmic localisation of HOX and PBX proteins and disrupted co-localisation patterns between HOX and PBX. **C** Colocalization of HOX and PBX proteins and colocalization of HOX/PBX dimers in the nucleus was quantified using ImageJ. Both colocalization of HOX and PBX proteins and localisation of HOX/PBX dimers in the nucleus were lost following HTL-001 treatment

To assess the specificity of HOX/PBX targeting by HTL-001 (i.e. target engagement) as an indicator of dimer disruption, we used a co-localisation immunofluorescence assay. HOXA1, HOXB5, HOXC4 and HOXC9 were chosen as a panel of HOX proteins that are most commonly upregulated in GBM tissues and cell lines that were used in this study. Additionally, these HOX proteins are known interactors of PBX1 and would be able to clearly show potential disruptive effects of HTL-001. U87 and LN18 cells were treated with the IC_50_ dose of HTL-001 for 2 h. Anti-HOX and anti-PBX primary antibodies were used to stain HOX and PBX proteins and fluorescent-labelled and Fluorescent-labelled secondary antibodies were used to visualise the cellular localisation of the proteins in question, Fig. 4. In untreated cells, HOX and PBX proteins remained localised throughout the cell, with an increased co-localisation intensity in the nucleus, where HOX-PBX dimers would be transcriptionally active. In cells treated with HTL-001, the co-localisation of HOX and PBX proteins decreased. There was markedly lower HOX protein localisation in the nucleus and an increased HOX protein localisation in the cytoplasm when cells were treated with HTL-001. This is confirmed by intensity graphs which quantify HOX and PBX proteins in the nucleus and show their nuclear co-localisation in untreated cells (Fig. 4C). This localisation changes following exposure to HTL-001 to become more cytoplasmic with dysregulated co-localisation patterns between HOX and PBX. This would imply that HOX-PBX dimer formation is more prominent in the nucleus where this dimer is transcriptionally active, and it is this nuclear interaction of HOX and PBX that HTL-001 is effective in disrupting.

#### HTL-001 induces the activation of apoptotic pathways mediated by cFOS, DUSP1 and EGR1

In most solid tumours treated with the prototype HOX/PBX inhibitor HXR9 cell death is mediated by apoptosis which we also demonstrated in GBM and has been shown to be activated, at least in part, from a rapid upregulation in cFOS expression To assess the apoptotic pathways that were activated from disrupting HOX/PBX dimers using HTL-001, the protein expression of cFOS, DUSP1 and EGR1 was quantified using western blot analysis. The presence of cFOS, DUSP1 and EGR1 was quantified in cells treated with the IC_50_ of HTL-001 for 2 h, with β-actin as a loading control. cFOS, DUSP1 and EGR1 showed significant upregulation in protein expression in cells treated with HTL-001, implying that HTL-001 induced apoptosis is likewise mediated by elevated levels of cFOS, DUSP1 and EGR1 that in turn activates pro-apoptotic pathways and/or inhibits anti-apoptotic pathways (Fig. 2D).

#### HTL-001 causes calpain-mediated apoptosis-inducing factor (AIF) release and translocation to the nucleus that induces caspase-independent apoptosis in glioblastoma cells

To determine if caspase-dependent or independent pathways of apoptosis are activated in HTL-001 treated cells, the presence and behaviour of the end-point caspase, caspase 3, was investigated. By western blot analysis, the presence of pro-caspase 3 (inactive form) and cleaved caspase 3 (active form) was quantified in cells treated with HTL-001 for 2 h.

There was a lack of cleaved caspase (Fig. 2E) and no significant increase in activated (Fig. 2G) caspase 3 protein after 2 h of HTL-001 treatment.

We next investigated the expression of AIF, a mitochondrial inter-membrane flavo-protein that is the main mediator of caspase-independent apoptosis; upon activation, AIF translocates from the mitochondria to the nucleus to induce chromatin condensation and DNA fragmentation. Western blot analysis of AIF protein in mitochondrial and nuclear fractions of glioblastoma cells treated with the IC_50_ of HTL-001 for 2 h, showed a significant elevation and activation of AIF protein in the nucleus after HTL-001 treatment (Fig. 2F).

AIF release from the mitochondria and translocation to the nucleus has been indicated in previous studies to be an effect of calcium activated calpains which cleave and AIF translocation to the nucleus. Calpain activity after HTL-001 treatment was assessed using the Calpain-Glo Protease assay. HTL-001 induced calpain activity in glioblastoma cells with an increase in calpain activity approximately 700% over control level (Fig. 2H). These results confirm that the prominent mechanism of HTL-001 induced apoptosis is via a caspase-independent, calpain mediated activation of AIF.

#### Systemic HTL-001 causes anti-tumour effects in human xenograft and subcutaneous and orthotopic murine GBM models

**Fig. 5:**
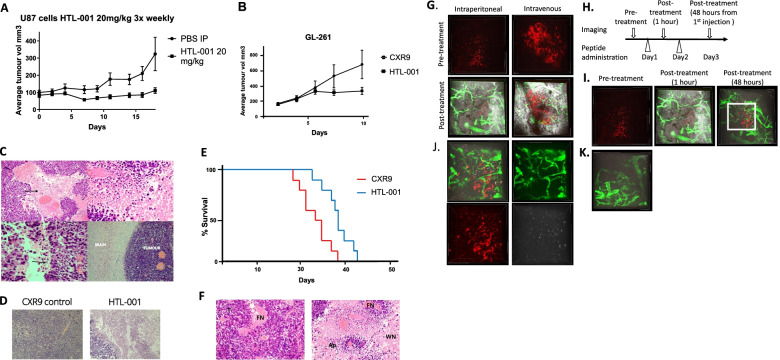
A Systemic treatment of U87-MG flank tumours with HTL-001**.** 100ul of live U87-MG cells (1e6 cells) in 50% matrigel were injected subcutaneously in the right flank of BALB c nude mice (*n* = 8 per group). When tumours were between 30-60mm3 in size (approx. day 10) the animals received an intraperitoneal injection of HTL-001 at a single site (IP), a maximum of three times in a week (1–2-day part), for four consecutive weeks. HTL-001 treatment resulted in significant disease stability compared to PBS controls, unpaired student t- test *P* = 0.0052 **B** Comparing the efficacy of HTL-001 treatment against the CXR9 control in a subcutaneous GL-261 glioblastoma model in mice GL-261 cells were subcutaneously inoculated into the right flanks of 6-week-old C57BL/6 mice. Once the tumour grew to 100 mm^3^, mice were randomized into groups receiving either CXR9 or HTL-001 (30 mg/kg) by intraperitoneal injection three times a week until the experimental endpoint. Tumour volume was measured every 2–3 days. Tumours treated with HTL-001 were significantly smaller compared to tumours treated with CXR9 (*P* = 0.001186). **C)**Tissue was resected from tumours treated with either CXR9 or HTL-001 and sections evaluated. Top left: low power view (magnification 40X) of tumour treated with HTL-001 with large areas of necrosis, as indicted by the arrow. Top right: High power view (magnification X200) of tumour treated with HTL-001 with evidence of apoptosis and necrosis. Bottom left: High power view (magnification X200) of tumour treated with HTL-001 with areas of apoptosis, as indicated by the arrow. Bottom right; Tumour from orthotopic model with adjacent normal brain wherein no effect of treatment can be seen (magnification X40). **D)** Areas of apoptosis (Ap) and necrosis (Ne) were observed in tumour tissue treated with HTL-001, but not in tissue treated with CXR9. **E** GL-261 cells were intracranially inoculated by stereotactic injection into 6-week-old C57BL/6 mice. After 7 days, tumour-bearing mice were IP treated with either CXR9 or HTL-001 (30 mg/kg) three times a week until the experimental endpoint. Kaplan–Meier analysis showed that mice treated with HTL-001 survived significantly longer than mice treated with CXR9 (P = 0.0078 via log-rank test). **F** Tissue was resected from tumours treated with either CXR9 or HTL-001 and evaluated after H&E staining, 40X magnification shown. Top. Tumour from CXR9 control mice showed typical features of GL-261 with dense undifferentiated tumour (T) and areas of spontaneous focal necrosis (FN). Bottom; Tumour from HTL-001 treated mouse showed extensive apoptosis (Ap) and widespread necrosis (WN). **G-K)** Intravital imaging of peptide accumulation in the brain of NSG mice bearing RFP tumours after systemic delivery. FITC dextran shows perfused blood vessels. 100 ul of HTL-001 Alex 660 peptide (5 mg/ml in PBS) was administrated intraperitoneally on day 2, and the confocal images of same area were acquired on day 3. **G** Both intraperitoneal and intravenous injection showed efficient delivery of peptide (white) into brain. **H** The scheme of experimental setup for C. **I** Time-course imaging showed the accumulation of peptide into tumour. **J** Inset of Fig.I **K** Non-tumour area did not show the peptide accumulation

We initially tested the in vivo efficacy of HTL-001 in subcutaneous models. Following inoculation of U87 cells, mice were treated either with 20 mg/kg HTL-001 or PBS thrice weekly via intraperitoneal injection. The HTL-001 treated mice showed a statistically significant retardation of tumour growth compared with PBS controls (Fig. 5A). An equivalent model in immunocompetent C57 black 6 mice of GL261 subcutaneous tumours, using a higher dose of 30 mg/kg thrice weekly HTL-001, yielded similar results, with significant tumour retardation compared this time to the same dosing of the control peptide CXR9 (Fig. 5B). Histological evaluation of all tumours indicated minor areas of well circumscribed spontaneous necrosis in control mice, but extensive apoptosis and necrosis in mice treated with HTL-001 (Fig. 5C and D). In both models, no significant drug-related toxicity was observed in the treated mice (Additional Fig. 5).

We next utilized a more clinically relevant orthotopic model of GBM. As this requires the test agent to cross the blood brain barrier, we formally evaluated the ability of HTL-001 to achieve this at the doses the mice would tolerate based on the initial experiments in subcutaneous models (above). After intravenous injection of Alexa 660 labelled HTL-001, high levels of signal, as expected, were observed within 30 min in the liver and kidneys with significant peptide retention in subcutaneous tumours. A low-level signal was observed in the brain of Alexa 660 HTL-001 treated mice, but not in the brains of untreated mice (Additional Fig. 6). To confirm this result, we used intravital imaging in NSG mice. In these experiments GBM12-RFP cells were implanted stereotactically into the right hemisphere, and 2 weeks later, cranial window surgery was performed. Following intraperitoneal or tail vein injection of HTL001-Alex 660, fluorescin isothiocyanate (FITC)-conjugated dextran was injected into the tail vein, and post-treatment images were acquired. To evaluate the accumulation of peptide in time-course, 100 µl of HTL001-Alex 660 (5 mg/ml in PBS) was administrated intraperitoneally on day 2, and the confocal images of the same area were acquired on day 3 (Fig. 5G-K).

Finally, we compared the efficacy of HTL-001 treatment against CXR9 control peptide in an orthotopic GL261 murine model. GL261 cells were intracranially inoculated by stereotactic (Additional Fig.7) injection into 6-week-old C57BL/6 J mice. After 7 days, tumour-bearing mice were treated IP with either CXR9 or HTL-001 (30 mg/kg) three times a week until the experimental endpoint. Kaplan–Meier analysis showed that mice treated with HTL-001 survived significantly longer than mice treated with CXR9 (*P* = 0.0078) (Fig. 5E). As with the subcutaneous GL261 model, we found evidence of widespread apoptosis and necrosis in tumours from mice treated with HTL-001 but not CXR9, as predicted by our in vitro data. (Fig. 5F).

## Discussion

There have been no significant advances in GBM treatment for over a decade. *HOX* gene dysregulation is now a widely recognised phenomenon in solid and liquid malignancies and has critical roles in mediating several hallmarks of cancer [35–38]. As such, *HOX* genes are considered to be potential biomarkers and therapeutic targets. However, their high degree of functional redundancy severely limits options for drug design. In this study, we have focused on HOX gene dysregulation in GBM as a disease with a high therapeutic unmet need, which is already associated with aberrations of individual HOX genes and contains CSCs that show highly elevated HOX gene expression.

Few studies have examined the expression of all 39 HOX genes and cofactor genes in individual GBM tumours. We approached this through public databases and found higher expression of all HOX genes in GBM tissue compared to normal tissue, with the exception of HOXD1.

We found that HOX genes from all 4 clusters were expressed in all GBM cell lines evaluated, with genes from the HOXA and HOXD clusters most highly overexpressed. Levels of expression were further elevated in the CSC counterparts of specific cell lines generated through serum deprivation and growth factor stimulation. This was also present in patient-derived CSCs, which concurs with previous findings that *HOX* genes play a role in the formation and maintenance of glioma CSCs [39–42], and is not simply a result of in vitro culture. Taken together with previous studies, our findings indicate that the *HOX* genes A9, A10, C4 and D9 are the strongest candidates for biomarkers and treatment targets for GBM and GBM CSCs. To overcome functional redundancy across HOX genes, our therapeutic strategy has been directed at HOX protein functions as transcription factors. By themselves, they have modest transcriptional activity. but this is markedly enhanced by binding to cofactors, particularly *PBX1-3*. We have previously shown that disrupting HOX/PBX dimers with a peptide mimicking the highly conserved hexapeptide motif in HOX proteins, that mediates binding to PBX, leads to rapid apoptosis across a wide range of cancers [27]. Subsequent studies with systematic amino acid substitutions in HXR9 gave rise to a more potent peptide inhibitor of the HOX/PBX interaction, HTL-001.

We found that incubation of GBM cell lines for only 2 h with HTL-001 led to rapid cell death. This effect was further enhanced in CSCs both in a naturally occurring CD133 + cell subpopulation in KNS42 cells, and also neurosphere forming artificial CSCs that show elevated HOX gene expression. Notably there was no correlation between methylation status of the MGMT promoter or specific mutational profiles, and either HOX gene expression or sensitivity to HTL-001; both important factors in resistance to chemotherapy and radiotherapy, respectively [43, 44]

The small size and large number of potential HOX/PBX dimer combinations does not allow a direct assay of their disruption, but a co-localisation immunofluorescence assay showed that HTL-001 treatment results in markedly reduced signal in the nuclei where these dimers would be transcriptionally active. Like HXR9, HTL-001 induces rapid upregulation of cFOS, DUSP1 and EGR1 [27]. cFOS is able to induce apoptosis via the AP1 transcriptional activator to activate Fas ligand expression that binds and activates Fas receptor-induced apoptosis [45]. DUSP1 is able to dephosphorylate and inactivate protein kinases of MAPK signalling necessary for cell proliferation [46]. EGR1 activates apoptosis in several cancers via pro-apoptotic gene activation.

The lack of caspase 3/7 activation indicates the activation of caspase-independent apoptosis. Calpains activate AIF which mediates caspase-independent apoptosis by translocating to the nucleus to cause chromatin condensation and DNA fragmentation. Caspase-independent apoptosis was confirmed by an increase in calpain expression alongside increased mitochondrial and translocated AIF expression.

Importantly, we also tested the in vivo effects of HTL-001 in a series of models. The initial biodistribution studies of Alexa660-labelled HTL-001 suggested the peptide could cross the blood brain barrier in non-cancer bearing mice and those harbouring intracranial tumours. Intravital imaging in mice with orthoptic glioma subsequently confirmed the accumulation of HTL-001 after intraperitoneal injection. Systemic delivery of HTL-001 resulted in control of subcutaneous murine and human xenograft tumours, and improved survival in a murine orthoptic model. The anti-tumour effect of HTL-001 in vivo was reduced compared to the effects seen in vitro due to the need of HTL-001 to cross the blood brain barrier to exert its effects; despite this, histological analysis of post treatment resected tumours showed widespread apoptosis. The necrosis observed may additionally have reflected the anti-angiogenic effects of HTL-001.

## Conclusions

HOX gene dysregulation is highly prevalent in GBM tumours and may form the basis of novel therapeutic strategies. Targeting post-translational protein–protein interactions of HOX protein binding with cofactors, and not individual HOX gene directed approaches, overcomes the functional redundancy which has proved limiting to date. HOX/PBX dimer disruption by HTL-001 leads to rapid apoptosis of both GBM tumours and CSCs. This is a novel therapeutic approach for a disease area of highest unmet need.

## Supplementary Information

**Additional file1:** 

**Additional file 2:** 

**Additional file 3:** 

**Additional file4:** 

**Additional file 5:** 

**Additional file 6:** 

**Additional file 7:** 

**Additional file 8:** 

## Data Availability

All data generated or analysed during this study are included in this published article [and its supplementary information files].
