# Supplementary figures and images for: HOX and PBX gene dysregulation as a therapeutic target in glioblastoma multiforme
Source: BMC Cancer. 2022 Apr 13;22:400. doi: 10.1186/s12885-022-09466-8 (PMC9006463; doi:10.1186/s12885-022-09466-8)

## Slide 1
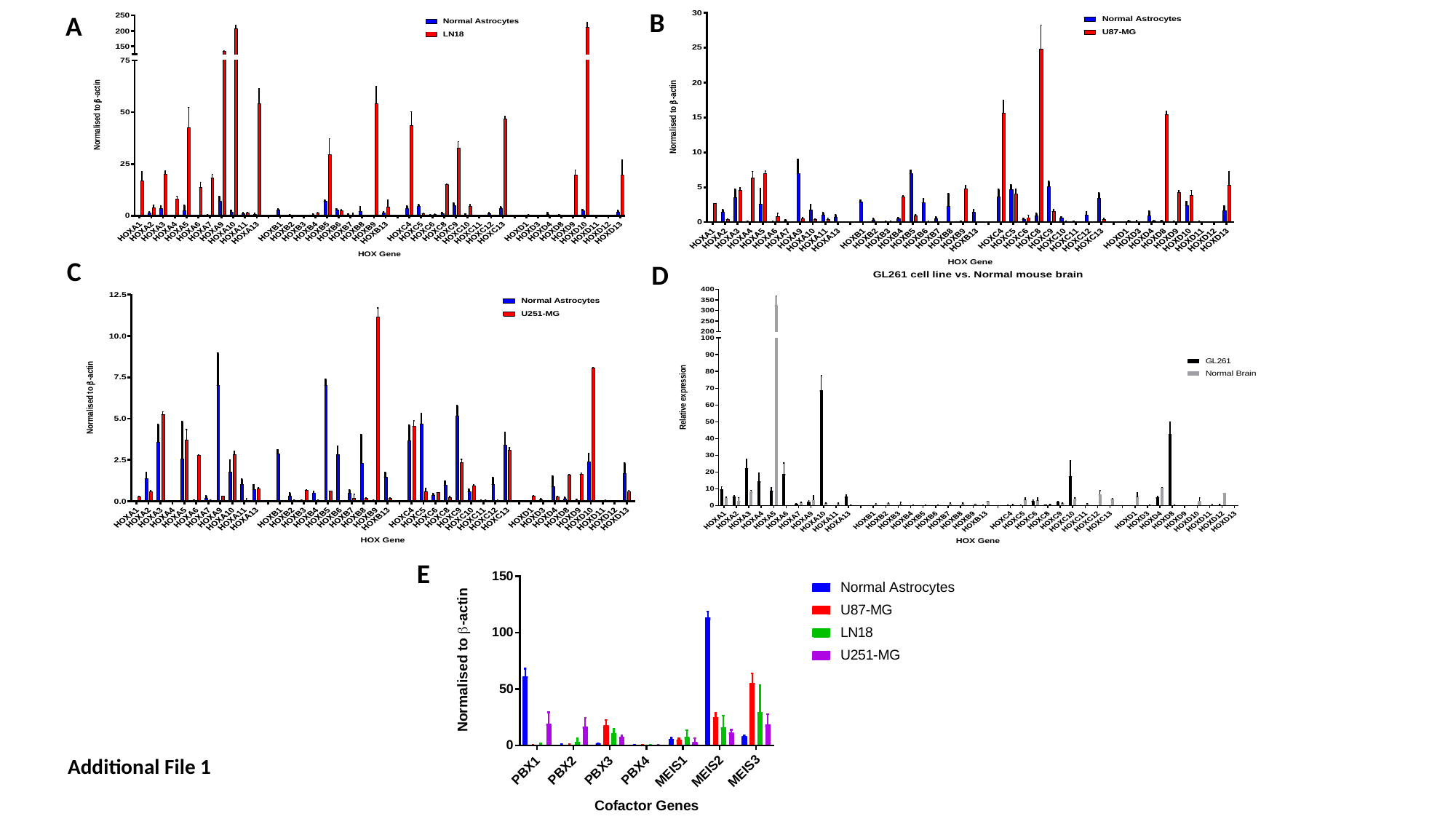

B
A
C
D
E
Additional File 1

Supplement: Supplementary file 1 — Additional file1: [file 12885_2022_9466_MOESM1_ESM.pptx]

## Slide 1
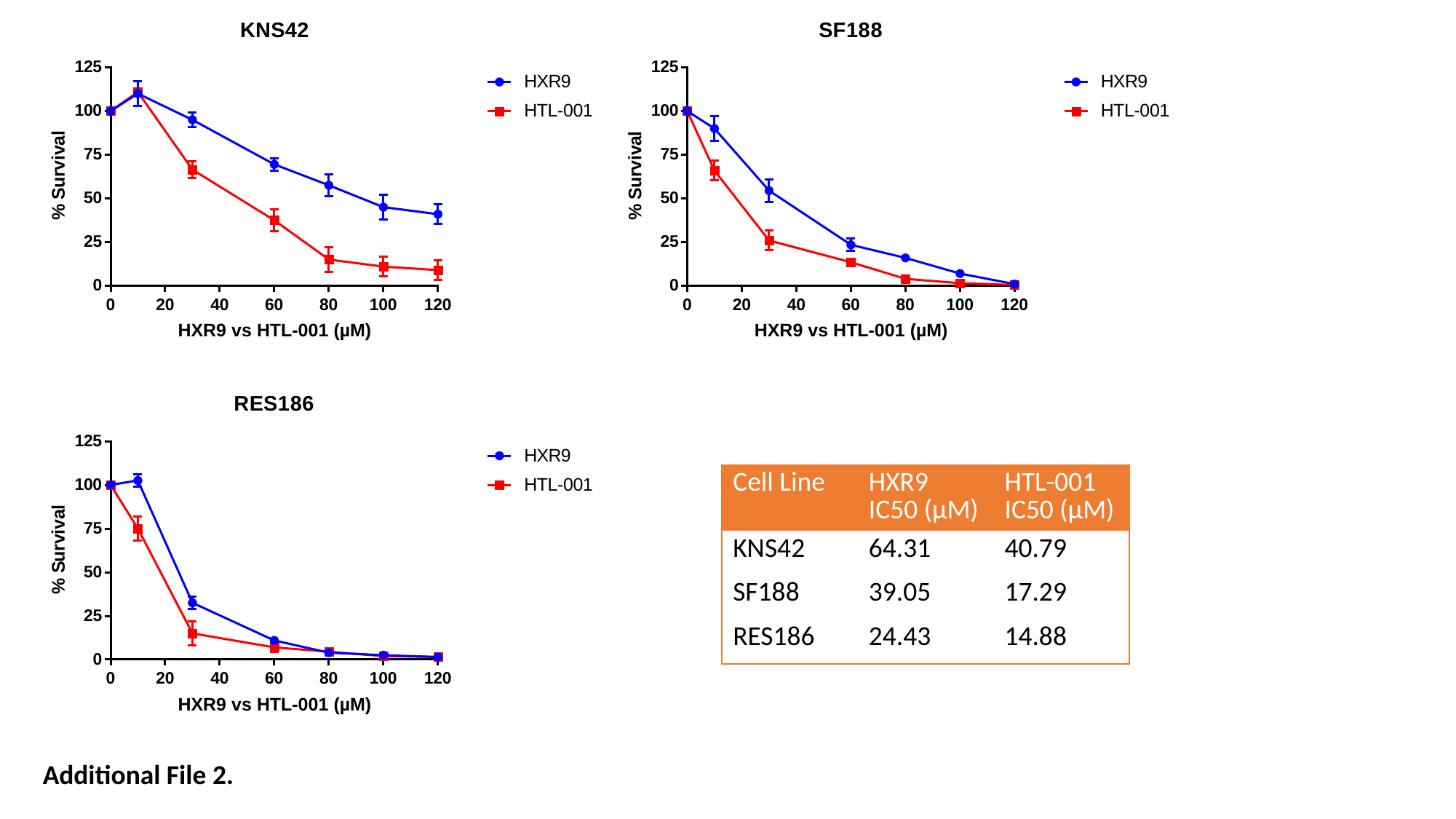

| Cell Line | HXR9 IC50 (µM) | HTL-001 IC50 (µM) |
| --- | --- | --- |
| KNS42 | 64.31 | 40.79 |
| SF188 | 39.05 | 17.29 |
| RES186 | 24.43 | 14.88 |
Additional File 2.

Supplement: Supplementary file 2 — Additional file 2: [file 12885_2022_9466_MOESM2_ESM.pptx]

## Slide 1
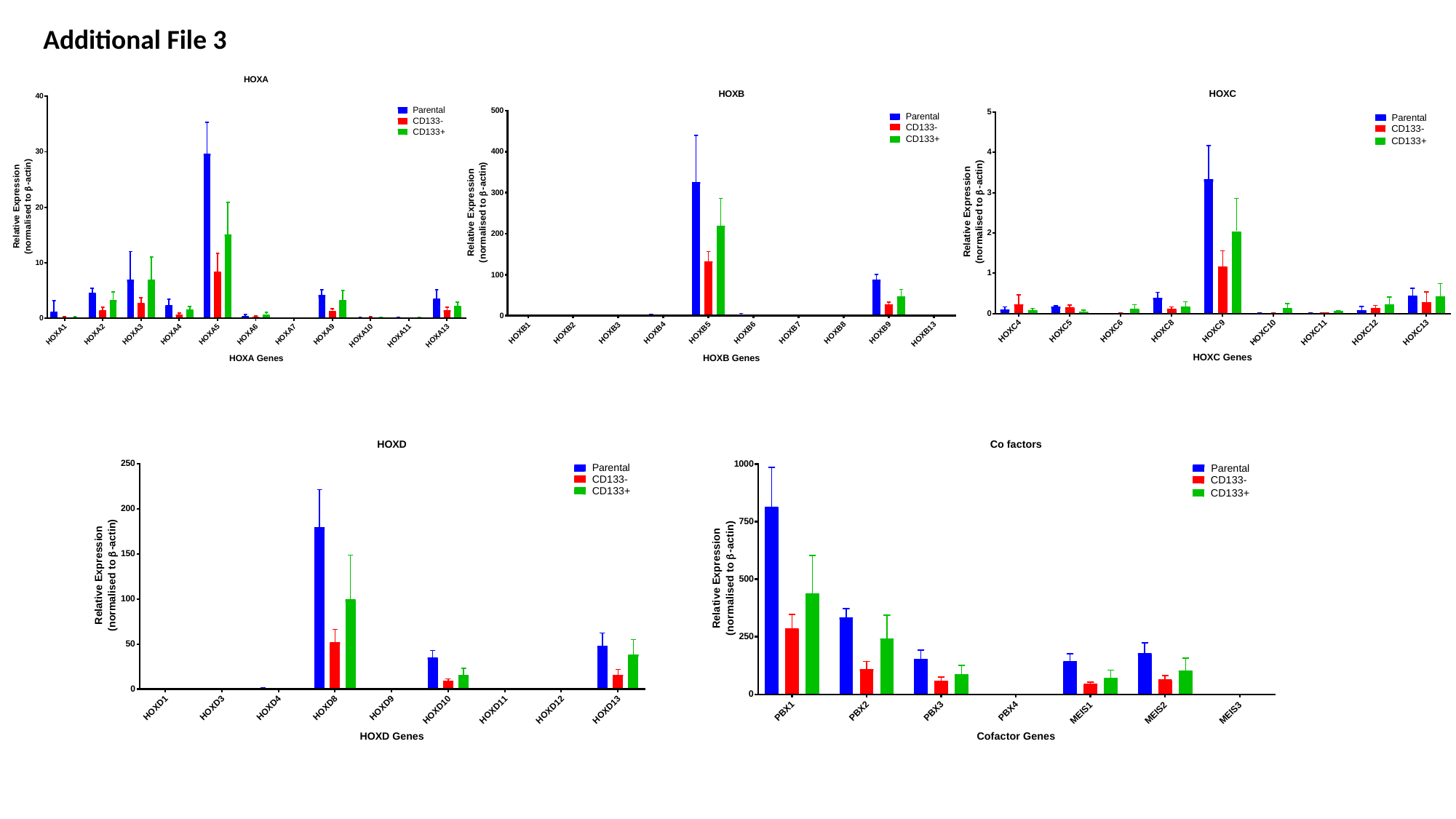

Additional File 3

Supplement: Supplementary file 3 — Additional file 3: [file 12885_2022_9466_MOESM3_ESM.pptx]

## Slide 1
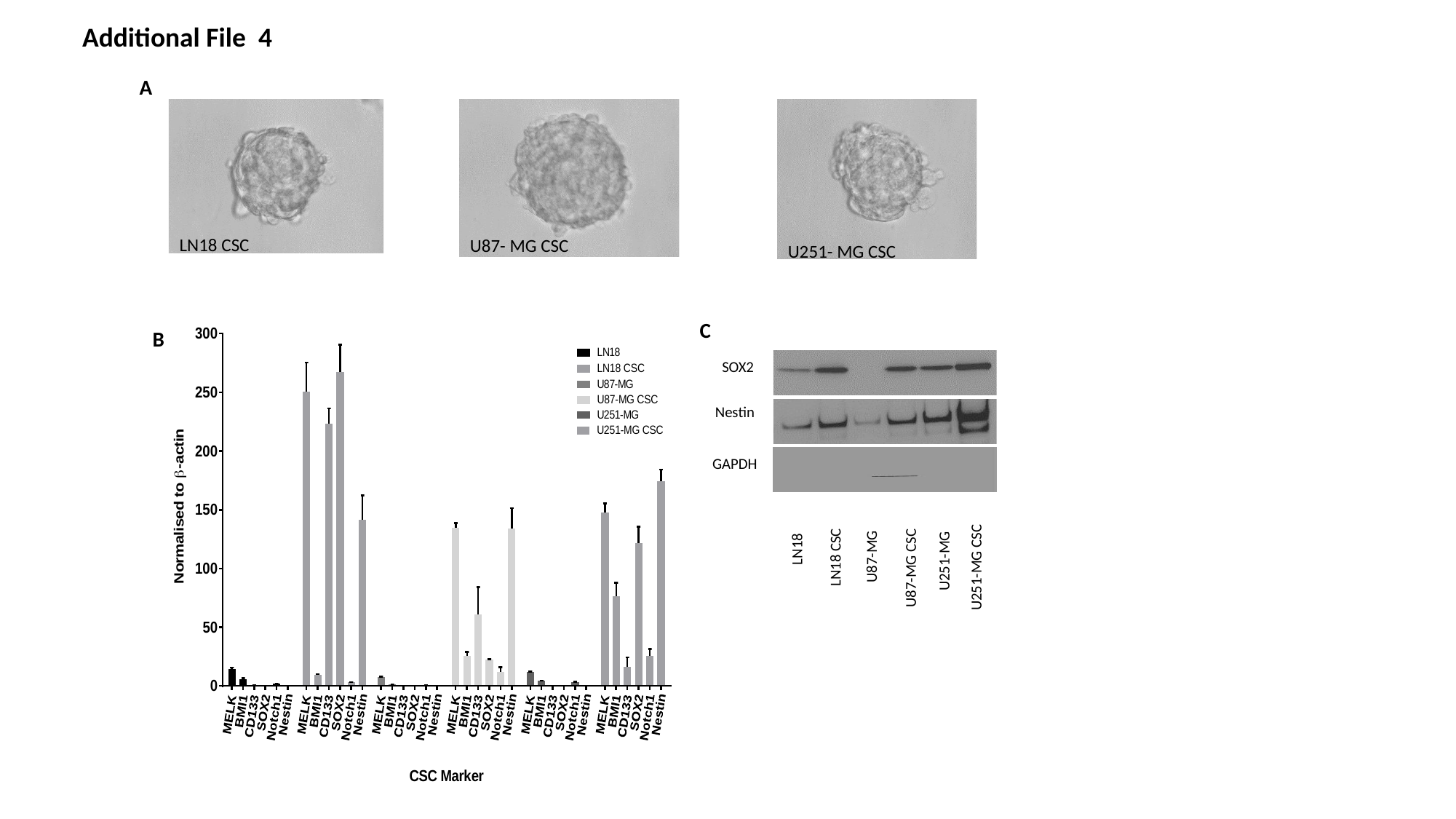

Additional File 4
A
LN18 CSC
U87- MG CSC
U251- MG CSC
C
B
SOX2
Nestin
GAPDH
LN18
LN18 CSC
U87-MG
U87-MG CSC
U251-MG
U251-MG CSC

Supplement: Supplementary file 4 — Additional file4: [file 12885_2022_9466_MOESM4_ESM.pptx]
