# Supplementary material for: HOX and PBX gene dysregulation as a therapeutic target in glioblastoma multiforme
Source: BMC Cancer. 2022 Apr 13;22:400. doi: 10.1186/s12885-022-09466-8 (PMC9006463; doi:10.1186/s12885-022-09466-8)
Supplement: Supplementary file 5 — Additional file 5: [file 12885_2022_9466_MOESM5_ESM.pptx]

## Slide 1
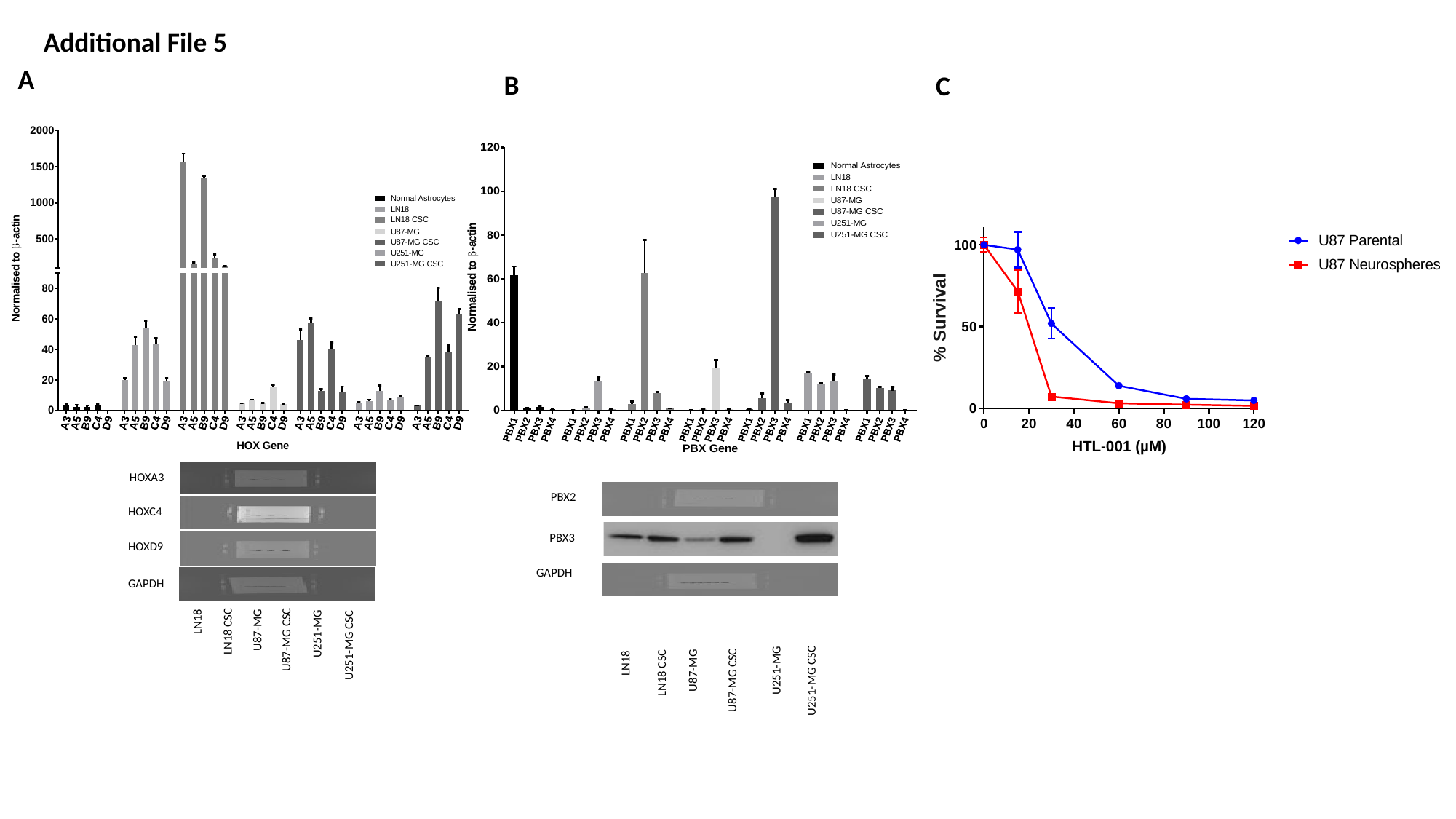

Additional File 5
A
B
C
HOXA3
HOXC4
U87-MG CSC
LN18 CSC
LN18
U87-MG
U251-MG
U251-MG CSC
HOXD9
GAPDH
PBX2
PBX3
GAPDH
LN18
LN18 CSC
U251-MG
U87-MG
U87-MG CSC
U251-MG CSC
