# Supplementary material for: HOX and PBX gene dysregulation as a therapeutic target in glioblastoma multiforme
Source: BMC Cancer. 2022 Apr 13;22:400. doi: 10.1186/s12885-022-09466-8 (PMC9006463; doi:10.1186/s12885-022-09466-8)
Supplement: Supplementary file 6 — Additional file 6: [file 12885_2022_9466_MOESM6_ESM.pptx]

## Slide 1
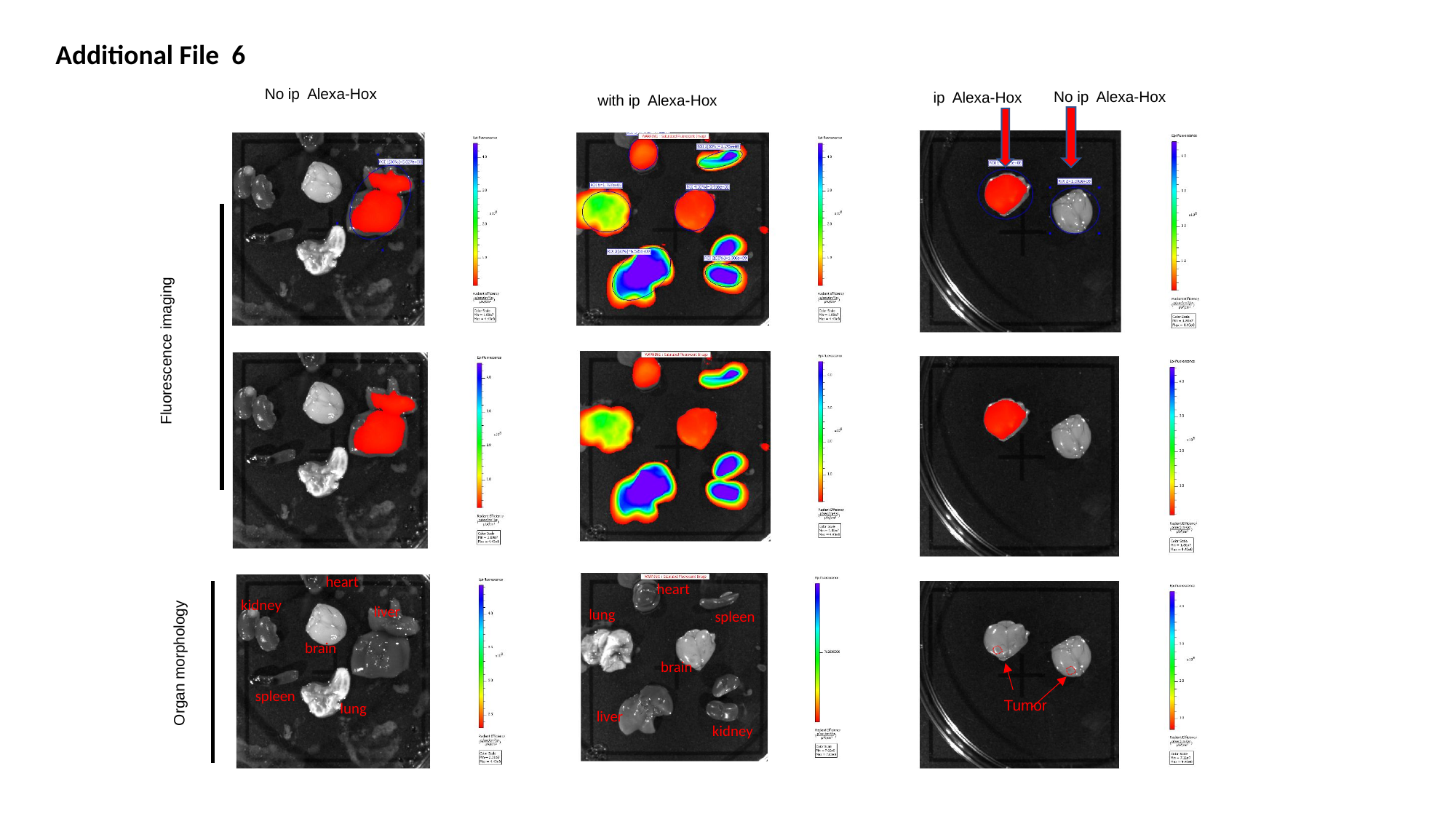

Additional File 6
No ip Alexa-Hox
No ip Alexa-Hox
ip Alexa-Hox
with ip Alexa-Hox
Fluorescence imaging
heart
heart
kidney
liver
lung
spleen
brain
Organ morphology
brain
spleen
Tumor
lung
liver
kidney
