# Supplementary material for: HOX and PBX gene dysregulation as a therapeutic target in glioblastoma multiforme
Source: BMC Cancer. 2022 Apr 13;22:400. doi: 10.1186/s12885-022-09466-8 (PMC9006463; doi:10.1186/s12885-022-09466-8)
Supplement: Supplementary file 7 — Additional file 7: [file 12885_2022_9466_MOESM7_ESM.pptx]

## Slide 1
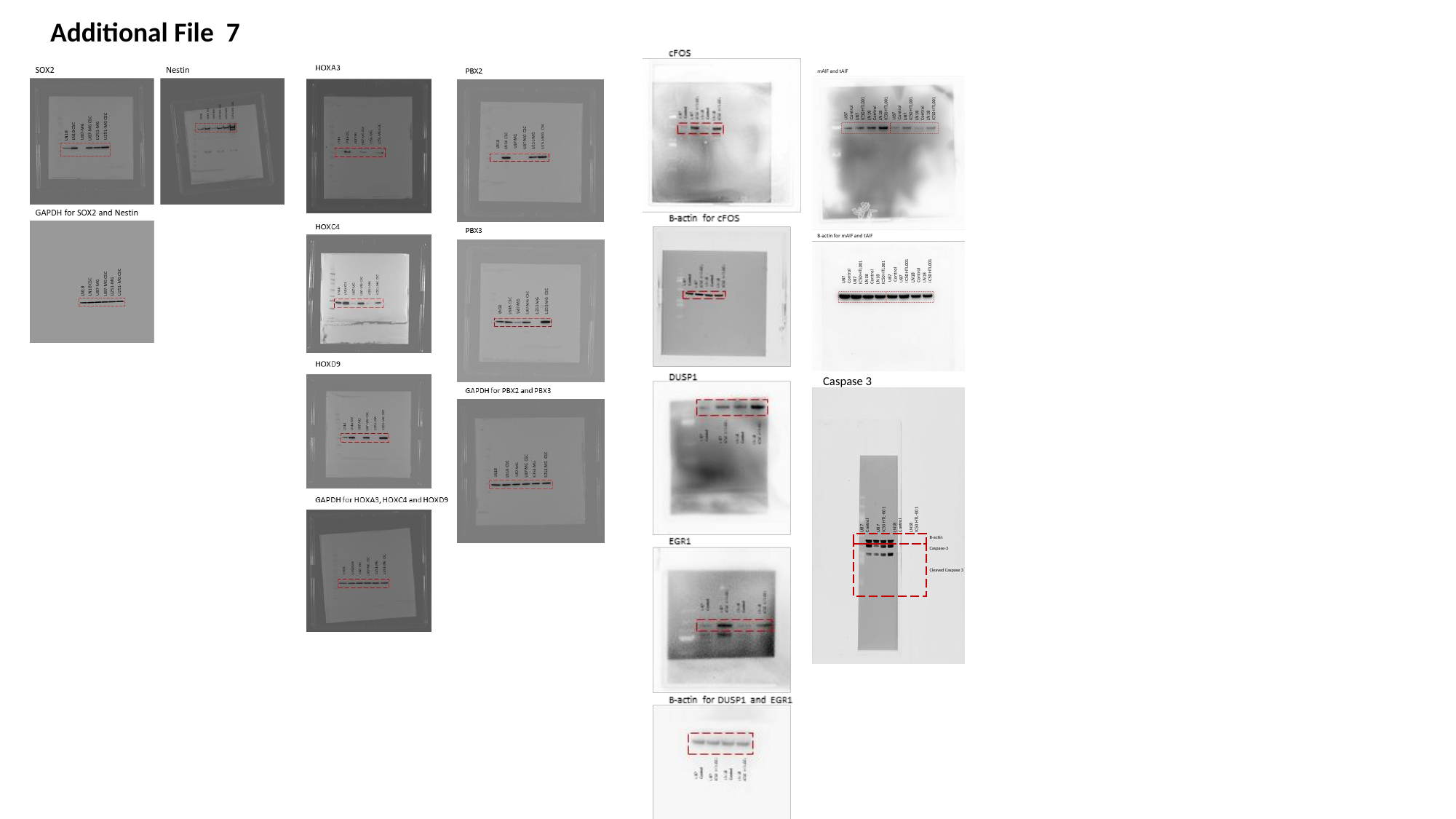

Additional File 7
Caspase 3
U87
Control
U87
IC50 HTL-001
LN18
Control
LN18
IC50 HTL-001
Β-actin
Caspase-3
Cleaved Caspase 3
