# Supplementary material for: HOX and PBX gene dysregulation as a therapeutic target in glioblastoma multiforme
Source: BMC Cancer. 2022 Apr 13;22:400. doi: 10.1186/s12885-022-09466-8 (PMC9006463; doi:10.1186/s12885-022-09466-8)
Supplement: Supplementary file 8 — Additional file 8: [file 12885_2022_9466_MOESM8_ESM.docx]

**Additional Materials and Methods**

**MTS cell survival assay**

Cells were seeded at a density of 10,000 cells per well in 96-well plates for 24 hours at 37°C. Media or drug was added to wells in replicates of eight for 2 hours. Cell Titre 96® AQ_ueous_ One Solution Reagent (Promega, UK; diluted 1:10) was added to each well and incubated for 2 hours at 37°C before reading the Optical Density (OD) absorbance readings on the GloMax® Microplate Reader (Promega, UK) at wavelength 492nm. The % cell survival in each treatment was calculated relative to un-treated cells. The drug IC_50_ values were determined using GraphPad Prism 8 software and interpolated using non-linear regression analysis of [inhibitor] vs response in a four-parameter curve fit model.

**Annexin V / 7-AAD staining of cell lines for cell viability**

Cells were seeded at a density of 300,000 cells per well in 6-well plates for 24 hours at 37°C. Media or drug (HTL-001) was added to wells and incubated for 2 hours before being harvested with trypsin and pelleted by centrifugation. Apoptotic cell death was determined using the Annexin V – PE Kit (BD Biosciences) according to the manufacturer’s instructions and analysed using MACSQuantify software on a MACSQuant flow cytometer (Miltenyi Biotec).

**RT-qPCR assays**

Total RNA was extracted from cells using the RNeasy® Plus Micro kit (Qiagen, UK) and cDNA was synthesised using Cloned AMV reverse transcriptase with oligo (dT)_20_ primers (Life Technologies, UK). QPCR was performed on a Stratagene Mx3000P instrument with Taqman Gene Expression Assays (targeting *DUSP1*, *EGR1* and *CFOS*) and the AgPath-ID™ One-Step RT-PCR kit, using the following program: 1 cycle (10 minutes at 45°C), 1 cycle (10 minutes at 95°C), followed by 40 cycles (15 seconds at 95°C and 45 seconds at 60°C). For analysis of stem cell markers (*CD133, MELK, SOX2, Nestin, BMI1* and *Notch1*) and for HOX Profiling for all 39 *HOX* genes and 7 TALE Cofactor genes, SYBR Green Jumpstart Taq Ready mix (Sigma, UK) was used with the following qPCR program: 1 cycle (10 minutes at 95°C), followed by 40 cycles (30 seconds at 95°C, 1 minute at 60°C and 1 minute at 72°C). Primer sequences for stem cell markers, *HOX* genes and *TALE* genes were designed and purchased from Sigma Aldrich. The 2^-∆CT^ relative quantitation method (36) was used to analyse the data. The primer sequences are shown in supplementary table 1.

**Western Blot**

Cells were seeded at a density of 300,000 cells per well in 6-well plates for 24 hours at 37°C. Media or drug was added to wells and incubated for 2 hours before being harvested with trypsin and pelleted by centrifugation. Total protein was extracted using RIPA Lysis and Extraction buffer (Thermo Fisher) supplemented with HALT protease and phosphatase inhibitor cocktail (Thermo Fisher). 20 µg of protein was run on a NuPAGE 4 to 12%, Bis-Tris Protein Gel (Thermo Fisher), blotted onto a PVDF membrane using using iBlot Tranfer (Thermo Fisher). Membranes were blocked in 1% skimmed milk before incubated overnight with the primary antibody at 4°C. The primary antibodies used were: AIF Antibody (Santa Cruz Biotechnology, sc-13116, 1:500), c-Fos Antibody (Cell Signalling Technlogy, 2250, 1:1000), Phospho-DUSP1/MKP1 Antibody (Cell Signalling Technology, 2857, 1:1000), EGR1 Antibody (Cell Signalling Technology, 4153, 1:1000), Caspase 3 Antibody (Proteintech, 19677-1-AP, 1:1000), β-Actin Antibody (Cell Signalling Technology, 8457, 1:2000). Membranes were then incubated with secondary antibodies for 1 hour at room temperature. Secondary antibodies were Anti-rabbit IgG HRP-linked Antibody (Cell Signalling Technology, 7074, 1:2000) and Anti-mouse IgG, HRP-linked Antibody (Cell Signalling Technology, 7076, 1:2000). SuperSignal™ West Femto Maximum Sensitivity Substrate (Thermo Fisher) and UVP BioDoc-It Imaging System was used to visualise the membranes.

**Caspase 3/7 and Calpain Activity Assays**

Cells were seeded at a density of 10,000 cells per well in white-walled 96-well plates for 24 hours at 37°C. Media or drug was added to wells in triplicates for 2 hours and caspase 3/7 activity was measured using the Caspase-Glo® 3/7 Assay System or Calpain-Glo™ Protease Assay (Promega, UK), according to the manufacturer’s instructions

**Peptides**

All peptides were synthesised by Sigma (UK). HTL-001 (WYKWMKKAARRRRRRRRR) is a modified version of the original HOX/PBX peptide inhibitor, HXR9 (WYPWMKKHHRRRRRRRRR), containing a number of amino acid substitutions that result in enhanced efficacy compared to the original version. CXR9 (WYPAMKKHHRRRRRRRRR) is a derivative of HXR9 that differs by a single tryptophan residue, which in HOX proteins is required for the interaction with PBX. This amino acid substitution renders CXR9 inactive with no cytotoxic effect in normal and cancerous cells, as shown in previous studies.

**Antibodies used in co-localisation Immunofluorescence**

The primary antibodies used were PBX1 Antibody (Cell Signalling Technology, 4342, 1:1000), anti-HOXA1 (Santa Cruz Biotechnology, sc-17146, 1:1000), anti‐HOXB5 (Santa Cruz Biotechnology, sc-81099, 1:1000) anti-HOXC4 (Santa Cruz Biotechnology, sc-398460, 1:1000) and anti-HOXC9 (Santa Cruz Biotechnology, sc-81100, 1:1000). Secondary antibodies were FITC‐conjugated goat anti-mouse IgG (Invitrogen, F2761, 1:50) and TRITC‐conjugated goat anti-rabbit IgG (Invitrogen, T2769, 1:50).
